# A Novel Lightweight CNN Architecture for the Diagnosis of Brain Tumors Using MR Images

**DOI:** 10.3390/diagnostics13020312

**Published:** 2023-01-14

**Authors:** Kamireddy Rasool Reddy, Ravindra Dhuli

**Affiliations:** School of Electronics Engineering (SENSE), VIT-AP University, Amaravati 522237, India

**Keywords:** brain tumors, convolutional neural networks, fast-linking, magnetic resonance imaging, skull-stripping, spiking cortical model

## Abstract

Over the last few years, brain tumor-related clinical cases have increased substantially, particularly in adults, due to environmental and genetic factors. If they are unidentified in the early stages, there is a risk of severe medical complications, including death. So, early diagnosis of brain tumors plays a vital role in treatment planning and improving a patient’s condition. There are different forms, properties, and treatments of brain tumors. Among them, manual identification and classification of brain tumors are complex, time-demanding, and sensitive to error. Based on these observations, we developed an automated methodology for detecting and classifying brain tumors using the magnetic resonance (MR) imaging modality. The proposed work includes three phases: pre-processing, classification, and segmentation. In the pre-processing, we started with the skull-stripping process through morphological and thresholding operations to eliminate non-brain matters such as skin, muscle, fat, and eyeballs. Then we employed image data augmentation to improve the model accuracy by minimizing the overfitting. Later in the classification phase, we developed a novel lightweight convolutional neural network (lightweight CNN) model to extract features from skull-free augmented brain MR images and then classify them as normal and abnormal. Finally, we obtained infected tumor regions from the brain MR images in the segmentation phase using a fast-linking modified spiking cortical model (FL-MSCM). Based on this sequence of operations, our framework achieved 99.58% classification accuracy and 95.7% of dice similarity coefficient (DSC). The experimental results illustrate the efficiency of the proposed framework and its appreciable performance compared to the existing techniques.

## 1. Introduction

The brain plays a crucial role in every aspect of human activity but studying its clinical elements is very challenging due to the complexity associated with its structure and functionality. Behind many medical complications in the brain, tumors are observed to be the main reason. Usually, it is created in or around the brain due to the unconstrained development of irregular cells, which may spread to other parts [1]. Typically, brain tumors are classified into primary and secondary (metastatic). Primary tumors begin in the brain, while secondary brain tumors arise from other body regions such as lungs, breasts, kidneys, skin, etc., and migrate to brain tissues through the bloodstream [2]. 

Further, primary brain tumors can be categorized as either cancerous (malignant) or non-cancerous (benign). Non-cancerous tumors do not have any active cells; hence, they can be wholly restrained and treated by a surgical process. On the other hand, cancerous tumors have active cells proliferating and attacking other brain areas. These tumors cannot be cured under regular medication but may be controlled by radiotherapy/chemotherapy. The survival rate of victims of cancerous tumors is low compared to non-cancerous tumors, so early brain tumor detection is crucial. In this process, imaging modalities such as magnetic resonance (MR) imaging and computed tomography (CT) [3] play an essential role. MR imaging modality is preferred since it generates high-contrast images without ionizing X-rays [4].

Researchers have recently focused on automated detection methodologies to detect brain tumors from MR images. Among them, segmentation-based approaches are more popular. The primary task of segmentation is to separate the affected and non-affected regions of the tumor. In many scenarios, the tumor area is visually distinguishable. However, it is challenging to attain accurate segmentation due to tumor intensity, texture, size, shape, and location variations. Many authors introduced automated methodologies to identify tumors from MR images [5]. Artificial neural networks (ANN) and deep learning approaches are used widely since they efficiently characterize complex tasks [6]. Motivated by this, various methodologies have been developed in the recent past to detect and classify brain MR images [7]. Among them, we discuss a few noted studies.

Kale et al. [8] suggested a diagnosis approach for brain diseases using local binary patterns (LBP), steerable pyramid (SP), and back-propagation neural network (BPNN). Singh et al. [9] proposed a hybrid technique based on discrete wavelet transform (DWT), independent component analysis (ICA), and kernel support vector machine (k-SVM). Bahadureet al. [10] introduced a computer-aided diagnosis system with the help of watershed, fuzzy C-means (FCM), and Berkeley wavelet transform (BWT). Gokulalakshmi et al. [11] implemented an enhanced classification technique using K-means clustering, DWT, and support vector machine (SVM). Toğaçar et al. [12] designed a BrainMRNet architecture based on hyper-column approaches and attention modules. 

Neffati et al. [13] proposed a compulsive brain tumor identification system with the help of DWT, kernel principal component analysis (KPCA), and SVM. Wang et al. [14] introduced an automatic diagnosis approach for classifying brain tumors using stationary wavelet entropy and energy features along with SVM. Arunkumar et al. [15] developed an enhanced automated brain tumor detection system using K-means clustering and ANN. Toğaçar et al. [16] presented a novel deep-learning approach with the help of recursive feature elimination (RFE) and SVM. Chanu et al. [17] suggested a two-dimensional convolutional neural network (2D-CNN) system based on data augmentation. 

Lu et al. [18] utilized AlexNet with transfer learning to develop an automatic brain tumor classification system. Vishnuvarthanan et al. [19] implemented an unsupervised methodology for the segmentation of brain tumors using a self-organizing map (SOM) and fuzzy K-means (FKM). Hasan et al. [20] proposed a computer-aided methodology based on a modified gray-level co-occurrence matrix (MGLCM) and multi-layer perceptron (MLP) neural network. Nagarathinam et al. [21] introduced an automated computer-aided approach using genetic algorithm (GA) and adaptive neuro-fuzzy inference system (ANFIS) methods. Ahmadi et al. [22] suggested a deep learning approach based on brain tumor segmentation using a convolutional neural network (CNN) and robust principal component analysis (RPCA). Toufiq et al. [23] suggested a hybrid feature extraction approach for identifying brain tumors from MR images. 

Ginni Garg et al. [24] proposed a hybrid ensemble model for classifying brain tumors from MR images using stationary wavelet transform (SWT), GLCM, and a hybrid classifier. Pitchai et al. [25] developed an MR-based brain tumor segmentation model using FKM and ANN. Siyuan et al. [26] suggested an improved AlexNet and extreme learning machine (ELM) followed by a chaotic bat optimization (CBM) framework for identifying abnormal brain tumors from MR images. Mantripragada et al. [27] introduce a novel brain tumor segmentation, and classification framework based on deep neural networks (DNN) and adaptive fuzzy deformable fusion (AFDM) approaches. Amin et al. [28] employed a CNN framework to detect and classify brain tumors. Arpit Kumar Sharma et al. [29] a modified ResNet50 architecture along with an enhanced watershed (EWS) algorithm was presented to differentiate abnormal from normal brain MR images. Sarang Sharma et al. [30] designed a deep-learning framework for predicting MR-based brain tumors. Alsaif et al. [31] suggested a novel brain tumor classification model based on CNN.

From Table 1, we summarize the issues encountered in the existing approaches.

1. Traditional automatic detection approaches utilized conventional machine learning algorithms, whose performance depends on the choice of appropriate features and learning approaches.
2. Some classification methods employed wavelets for image analysis. However, they fail to acquire directional information; the selection of subbands and mother wavelets is also critical.
3. Some approaches use handcrafted features but are not robust to noise and exhibit poor discrimination.
4. The authors implemented some traditional CNN frameworks such as pre-trained CNN models with transfer learning to classify brain MR images in a few works. However, they demand a large number of parameters and high computational time.

To address the abovementioned problems, we suggested a new approach for identifying and classifying brain MR images using a fast-linking modified spiking cortical model (FL-MSCM) and lightweight CNN. 

### Significant Contributions

The significant contributions of this work are summarized as follows:

1. Skull-stripping is performed to enhance the robustness of the segmentation process by eliminating extra-meningeal mater (or dura mater) based on thresholding and morphological operations.
2. Image data augmentation is implemented to enhance the sufficiency and diversity of the training database by geometric transformation operators. By this, we significantly reduce the overfitting issues encountered during training progress.
3. We proposed a novel lightweight CNN architecture to detect high-level features from brain MR images. We can effectively minimize the parameters, including trainable and non-trainable, compared to the existing CNN models and automatically extract the significant features. Due to this, we limit the influence of human beings in the analysis of brain MR tumor images, which is the considerable benefit of the suggested CNN model.
4. Analyze the impact of various optimization algorithms (Stochastic gradient descent with moment (SGDM), Adam, Adagrad, AdaMax, Adadelta, Nadam, and RMSProp) during training of the CNN model with the help of K-fold cross-validation (KFC). It is the fundamental difference between the existing and proposed models.
5. The FL-MSCM is employed to separate the foreground (affected regions) and background (non-affected areas) from brain MR images, which can minimize issues of other traditional segmentation algorithms, such as the impact of noise, spurious blobs, and other imaging artifacts, by making each region as uniform as possible. Due to this, we improve the segmentation accuracy, which is a significant advantage of the presented FL-SCM technique.

The remaining part of the work is organized as follows: Section 2 represents the background of the CNN model. Section 3 illustrates the proposed technique and metrics to evaluate the performance of the models. Section 4 analyzes the outcomes and reasons behind the proposed method’s success and compares it with other state-of-the-art approaches. Section 5 discusses the conclusion of the present work.

## 2. Preliminaries

In this section, we discuss the background of deep learning and describe various layers used in the implementation of the proposed model in detail. Deep learning (DL) architectures can learn complex tasks by hierarchically constructing feature maps. CNN-based methods are more popular among the available DL models and have the following layers: convolutional, pooling, activation, batch normalization, fully connected (FC), and softmax, respectively.

### 2.1. Convolutional Layer

The convolutional layer plays a crucial role in classification. Typically, it produces many feature maps, F by convolving the input image with a set of filters in a sliding window manner as follows:

(1) $$F(u,v)=(B⊛C)(u,v)=\sum_{m}\sum_{n}B(u,v)C(u-m,v-n),$$

where ⊛ represents the convolution operator, B is the segmented image, C denotes the filter kernel, u and v are the indices of the generated feature map.

### 2.2. Batch Normalization Layer

It is also termed the batch norm and is mainly used to enhance the stability of a network by normalizing the features obtained from a convolutional layer, or FC layer. Typically, it lies between the convolutional and activation layer. The main advantages of this layer are:

1. Improving the training speed of the network.
2. Minimizing the internal covariance shift [32].
3. Reducing overfitting since it has slight regularization.

The entire process of the batch norm is described in Algorithm 1.
**Algorithm 1**. Batch normalization**Input:** Values of F over a mini-batch: $b=\left\{F_{1},F_{2},\ldots ,F_{K}\right\}$.  Parameters to be learned: γ,ξ. $\mu_{b}=\frac{1}{K}\sum_{j=1}^{K}F_{j},$                (2) $\sigma_{b}^{2}=\frac{1}{K}\sum_{j=1}^{K}{\left(F_{j}-\mu_{b}\right)}^{2},$           (3) $\hat{F_{j}}=\frac{F_{j}-\mu_{b}}{\sqrt{\sigma_{b}^{2}+\epsilon }},$                 (4) $b_{n}(F_{j})=\gamma \hat{F_{j}}+\xi ,$               (5) where γ represents scale; ξ illustrates shift; K is the number of feature inputs; μ and $\sigma^{2}$ are the mean and variance across the batch, b; ϵ is a constant, which is used to enhance the stability when $\sigma_{b}^{2}$ is too small. Output: $b_{n}\left(F_{j}\right)$

### 2.3. Activation Functions

**Figure 1:**
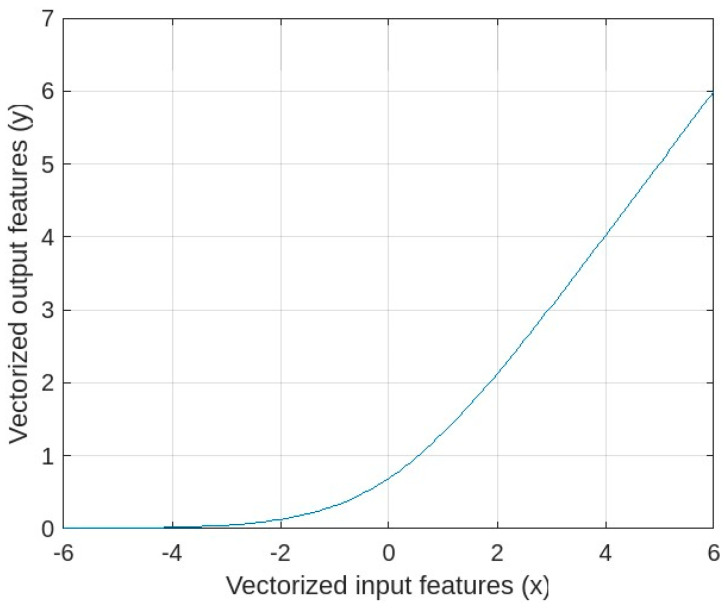
Softplus activation function.

Usually, activation functions are incorporated after the convolutional layer, establishing non-linearity in each neuron’s output. Due to this, the network will be able to learn many complex tasks. In this work, we utilized the softplus activation function, which is a smoothed version of rectified linear unit (ReLU) as shown in Figure 1. Mathematically the softplus function is defined as

(6) $$y=\log\left(1+e^{x}\right).$$

### 2.4. Pooling Layer

**Figure 2:**
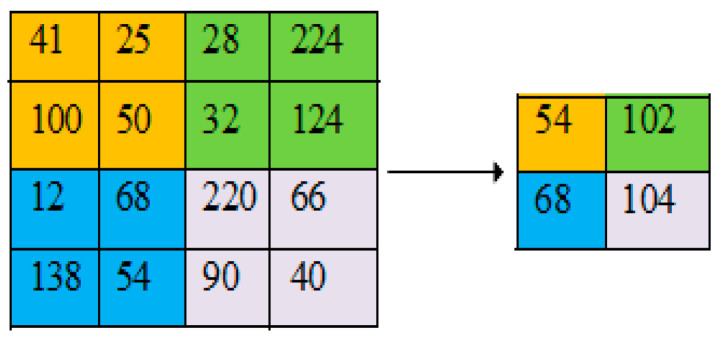
2 × 2 average pooling with stride 2.

**Figure 3:**
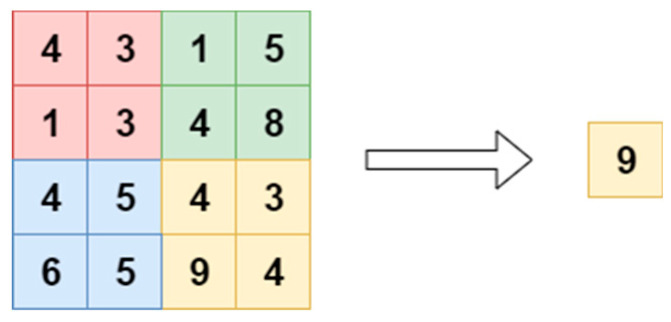
Global average pooling with stride 2.

The main goal of this layer is to scale down the spatial size of feature maps obtained from the preceding layers, minimizing the number of parameters to be learned and reducing computational time. Average pooling and max-pooling are the most frequently used approaches [33]. In our work, we utilized average and global average pooling (GAP), which is achieved by estimating the average value from each/entire region of the feature map, as shown in Figure 2 and Figure 3. Here, the main objective of the GAP is to yield one feature map for each corresponding classification task category, which avoids the overfitting problem. 

### 2.5. Softmax

Typically, the softmax is employed at the end of the neural network to transform the features into class probabilities. The softmax yields a value for each class based on the computation of probabilities given by

(7) $$P{\left(y\right)}_{i}=\frac{e^{f^{T}w_{i}}}{\sum_{j=1}^{M}e^{f^{T}w_{j}}},\;i=1,2,3,\ldots ,F$$

where f is the feature vector; T indicates the transpose operator; w illustrates the weight vector; P is the predicted probability of i-th class and finally, M represents the number of classes. Here, we have chosen M as 2 since we perform binary classification.

## 3. Materials and Methods

**Figure 4:**
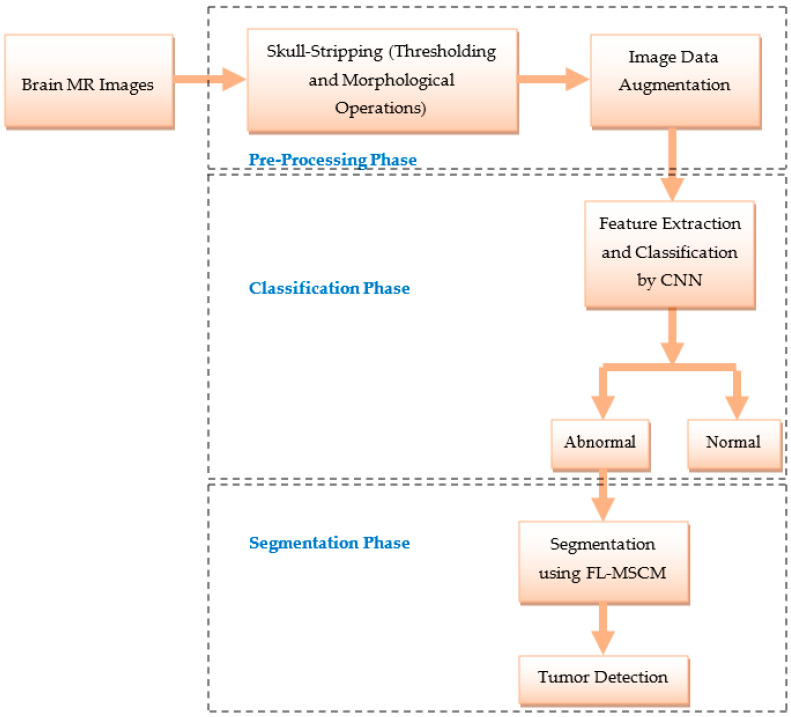
Flow diagram of the suggested brain tumor segmentation and classification approach.

The proposed system for identifying and classifying brain MR images is represented in Figure 4, and it includes the collection of the database, skull-stripping, image data augmentation, feature extraction and classification by CNN model, and tumor detection using FL-MSCM.

### 3.1. Database

To measure the effectiveness of the presented framework, we collected 60 normal and 125 abnormal T2-weighted brain MR images (glioma, metastatic adenocarcinoma, meningioma, sarcoma, and Alzheimer’s diseases) from a publicly available data source such as Harvard Medical School [34]. However, we cannot develop an effective diagnosis model based on this small sample size. Therefore, further, we generated augmented images with the help of rotation, translation, reflection, shearing, and scaling geometric transformation operations. Before implementing this step, we performed a skull-stripping process to improve the detection accuracy of the model.

### 3.2. Skull-Stripping

Skull-stripping is a significant preliminary stage in the analysis of biomedical images, which helps improve the effectiveness of brain tumor segmentation during the diagnosis of patients [35]. The main objective of this approach is to extract brain tissues by eliminating non-brain matters such as fat, skin, skull, etc. There have been numerous approaches [36]; thresholding and morphology-based procedures are more popular among them. Inspired by this, we proposed a combination of thresholding and morphological operations to achieve better skull-stripping.

1. Initially, we separate the image, I into two regions $R_{1}$ and $R_{2}$ over an intensity-level of [0,1,2,…,t−1], and [t,…,L]. Here, L is the number of intensity levels, usually an integer power of 2.
2. Obtain the binary image, B by setting the optimal thresholding value, $T_{opt}$ which is estimated by the following equations
  (8) $$H=\left(\frac{{\left(m_{1}-m_{2}\right)}^{2}}{s_{1}^{2}+s_{2}^{2}}\right),$$
  (9) $$T=\frac{H+\min\left(I\right)}{2},$$
  (10) $$T_{opt}=\frac{T}{255},$$
  where $m_{1}$, $m_{2}$ and $s_{1}^{2}$, $s_{2}^{2}$ represents the mean and variance of the regions over $R_{1}$, and $R_{2}$; T define the thresholding.
3. Construct a disk-shaped structuring element, $S_{d}$ with a required radius.
4. Eliminate the small peak objects from B using a simple area opening operation and then fill the regions with an image filling operation.
5. Employ the erosion operation on the outcome of step 3 with the defined $S_{d}$. Using this, we can eliminate small objects which appear in the binary image B.
6. Finally, the binary image obtained in step 5 is superimposed on the original image, I and replaces the non-binary region with zeros. With this process, the skull-free brain MR image is obtained, which improves the segmentation accuracy.

### 3.3. Image Data Augmentation

Deep learning heavily depends upon the massive amount of data to prevent overfitting. Overfitting is the phenomenon that occurs when a model learns a function with huge variance, which results in high performance on the training database, but fails to obtain high accuracy on the testing database. Hence, to mitigate this problem, we need to increase the number of samples in the given database. To meet this criterion, in this work, we employed data augmentation on skull-stripped images using geometric transformation techniques such as rotation, scaling, translation, and shearing along x- and y-directions, and reflection. Table 2 illustrates the configurations of the suggested augmented operators. We finally attained 540 normal and 1125 abnormal brain MR images with these operators. After that, we deployed the lightweight CNN model onto augmented images to predict the abnormality of brain MR images.

### 3.4. The Suggested Lightweight CNN Architecture

**Figure 5:**
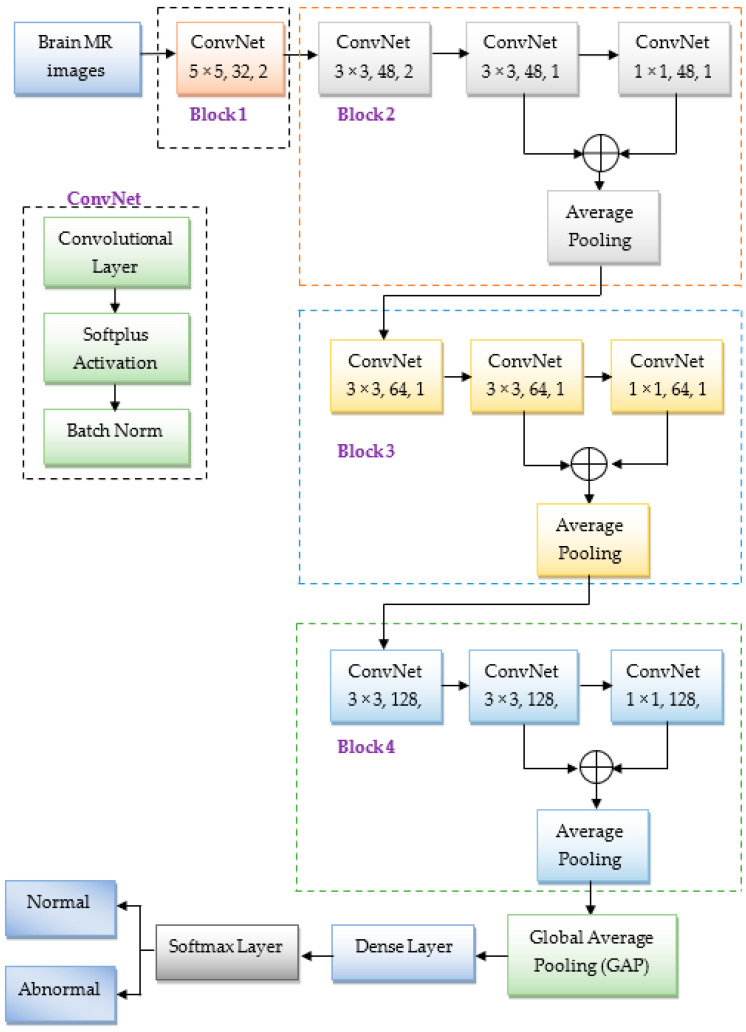
Architecture of the suggested CNN model.

In the literature, various conventional CNN frameworks [18,26,29,30] were discussed to identify the abnormality of brain MR images. However, they demand a large number of parameters to yield better accuracy, as results increase the computational complexity. Hence, we proposed a lightweight CNN architecture. With the help of our model, we can minimize the number of learning parameters and reduce the training speed without compromising the classification performance. It is the significant difference between the conventional and lightweight CNN models. The architecture of the presented CNN model is illustrated in Figure 5. The fundamental building block of our model is ConvNet, and it includes a convolutional layer, softplus activation function, and batch norm. The structure of the ConvNet is illustrated on the left side of Figure 5.

The proposed CNN model has four blocks, denoted by Blocks 1, 2, 3, and 4. The first block has only one ConvNet module. But the rest of the blocks have three ConvNet modules followed by a 2 × 2 average pooling with the stride of 2 and an adder operator to add the feature map values by point-to-point except the first block. The configurations of ConvNet in each block are as follows:

1. In the first block, the ConvNet module has 32 filters with a 5 × 5 kernel size, and the stride is 2. Here, the stride of 2 for the convolutional filter minimizes the input’s size to half, resulting in reduced computational complexity. Usually, the initial convolutional layers extract edge features; therefore, the stride of 2 will not significantly impact the model’s accuracy at initial convolutional layers.
2. Block 2 has three ConvNets, and they have 48 filters with a kernel size of 3 × 3, 3 × 3, and 1 × 1, and the strides of 2, 1, and 1, respectively. Similarly, blocks 3 and 4 contain three ConvNets with 64 and 128 filters. Each filter has a size of 3 × 3, 3 × 3, and 1 × 1, with a stride of 1. Here, the 1 × 1 convolutional filter is mainly used to minimize the computational requirements, i.e., reduce the dimensionality of the feature map. Due to that, the proposed CNN model required significantly fewer learnable parameters to train the model, as illustrated in Table 3. From this table, we observed that the total number of parameters is nearly 0.35 million. This number is much less than the other traditional CNN models discussed in the literature such as AlexNet [18,26], ResNet-50 [29], VGG-19 [30], etc. Hence, we called as a lightweight CNN.
3. In each ConvNet, we used a batch norm layer to improve the training speed and minimize overfitting.

At the end of block 4, we incorporated one GAP layer, a dense layer, and a softmax layer having two classes in sequence. Here, the GAP is used to compress the feature map by taking an average of each incoming feature map. After implementing the proposed CNN model, the resultant outcomes fed to the segmentation phase for identifying the infected area of abnormal brain MR images.

### 3.5. Segmentation

The main objective of segmentation is to improve diagnosis by automatically identifying suspicious patterns. However, it is a challenging task due to the artifacts, soft tissue boundaries, irregular shapes of brain tissues, etc. To address this, we developed a new brain tumor segmentation methodology termed fast-linking modified spiking cortical model (FL-MSCM), motivated by the work in [37].

#### 3.5.1. Modified Spiking Cortical Model (MSCM)

**Figure 6:**
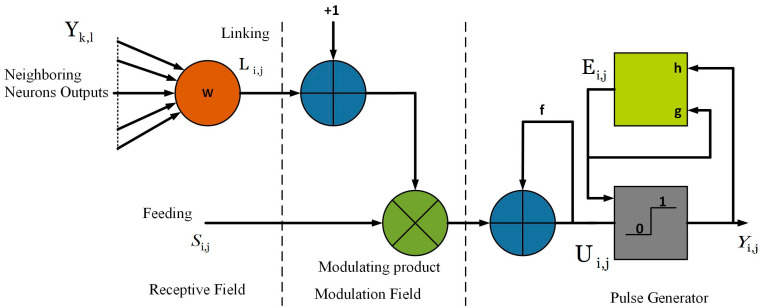
Flow diagram of the SCM model.

The spiking cortical model (SCM) [38] is derived from Eckhorn’s visual cortex model [39] and is developed especially for image processing applications such as segmentation, fusion, texture retrieval, etc. The functional flow graph of the SCM is illustrated in Figure 6, and it consists of a receptive field, a modulation field, and a pulse generator. In the receptive field, each (i,j)-th neuron has a feeding input $S_{i,j}$ and linking input $L_{i,j}$. In the modulation area, the membrane potential (internal activity), $U_{i,j}$ of the neuron is obtained by multiplying $S_{i,j}$ with $L_{i,j}$. Finally, the neuron fires and provides a pulse output, $Y_{i,j}$ when $U_{i,j}$ greater than threshold $E_{i,j}$. The equivalent mathematical expressions for this procedure are given below:

(11) $$L_{i,j}(n)=m_{L}\sum_{k,l}W_{i,j,k,l}Y_{k,l}(n-1),$$

(12) $$U_{i,j}(n)=fU_{i,j}(n-1)+S_{i,j}\left(1+\beta L_{i,j}(n)\right),$$

(13) $$Y_{i,j}(n)=\{\begin{array}{ll} 1 & U_{i,j}(n)>E_{i,j}(n)\\ 0 & \mathrm{else} \end{array},$$

(14) $$E_{i,j}(n)=gE_{i,j}(n-1)+h\times Y_{i,j}(n),$$

where (k,l) denotes the positions of neighboring neurons, n is the number of iterations, $W_{i,j,k,l}$, and $m_{L}$ represent the weight matrix and magnitude scaling factor of linking field, respectively, β is the linking strength, f is decay constant which always lies between 0 and 1. In our work, S is the input image and $S_{i,j}$ is the intensity value at (i,j) pixel location. 

In the conventional SCM model [38], to estimate E, an exponential decay function g is used, which results in slow computation. To speed up the process, we employed the MSCM approach with a linear decay mechanism to obtain the E value as follows

(15) $$E_{i,j}(n)=E_{i,j}(n-1)-\Delta +h\times Y_{i,j}(n),$$

where h is the threshold magnitude component and Δ ensures that the entire neuron threshold decays linearly.

From Equations (11)–(14), we note that the proposed approach has only one convolution term and two leaky integrators. It is the significant advantage of MSCM over pulse-coupled neural networks [40].

#### 3.5.2. Parameter Settings of MSCM

In the implementation of MSCM, the parameters are initialized as follows:

1. Firstly, the output, Y and internal activity, U are initialized as ‘zero’.
2. Threshold, $E_{i,j}(n)=1$.
3. Decay constant, f=0.2.
4. Magnitude scaling factor, $m_{L}=1$.
5. Threshold decay, Δ=0.02.
6. Due to the position invariant nature W can be determined by a 7 × 7 Gaussian filter with standard deviation ‘1′, which is utilized to estimate the precision level of the image pixel.
7. The threshold magnitude component, h is employed to ensure that each neuron will not fire more than once and is estimated using Equation (16).
  (16) $$h=\frac{\max\left(S\right)-\min\left(S\right)}{1-f}+\max\left(S\right)\left(1+\beta \sum_{k,l}W_{k,l}\right),$$
  where the linking strength, β is obtained by the following expression:
  (17) $$\beta =\frac{1}{1+e^{-G}},$$
  where $G=\sqrt{G_{x}^{2}+G_{y}^{2}}$ and $G_{x}$, $G_{y}$ are the central difference gradient of S along x- direction and y- direction.
8. The maximum number of iterations N can be determined as follows:
  (18) $$N=\left(\frac{\max\left(S\right)-T_{S}}{\Delta }+1\right),$$
  (19) $$T_{S}=\frac{T_{G}}{1-f},$$
  where $T_{G}$ is the gray-level thresholding of S, estimated from Otsu’s approach [41]. Here, the primary objective of thresholding is to calculate the number of iterations. For better segmentation, we apply the fast-linking algorithm to MSCM.

#### 3.5.3. Fast-Linking

Here, compared to normal linking [42], the neurons with similar stimuli respond quickly and synchronously. It mainly includes two loops:

1. Internal loop: Here, U and Y are repeated until Y does not vary.
2. External loop: Here, the function E is iterated.

**Figure 7:**
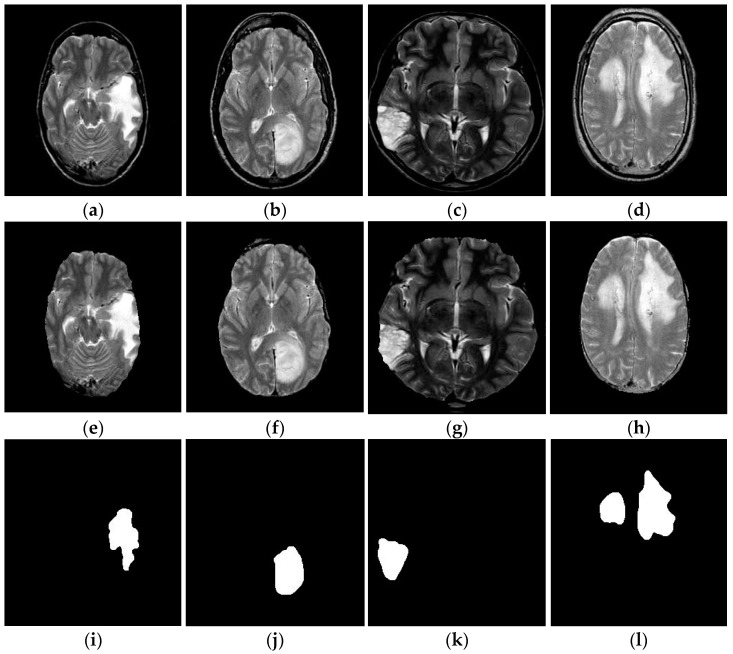
Outcomes of the proposed brain tumor segmentation: (**a**–**d**) Original brain tumor MR images. (**e**–**h**) Skull-stripped images. (**i**–**l**) Infected tumor region of the brain MR images by FL-MSCM.

The above process is depicted in Algorithm 2, and the corresponding outputs of FL-MSCM are shown in Figure 7i–l. This figure shows that the proposed segmentation approach significantly separated the tumor and non-tumor regions from skull-free brain MR images.
**Algorithm 2.** The fast-linking approach.n=0 **while**n≤N**do**  n=n+1  Update E by Equation (14)   **repeat**    $\hat{Y}=Y$,    Update L, U and Y by Equations (11)–(13),  **until**
$\hat{Y}==Y$ **end while**

### 3.6. Performance Metrics

The performance of the proposed model is evaluated using various well-known metrics such as true positive rate (TPR), true negative rate (TNR), positive predictive value (PPV), F-score, accuracy, and the area under the curve (AUC) [43]. TPR estimates the percentage of accurately identified abnormal brain MR images, while TNR measures the percentage of precisely recognized normal brain MR images. PPV calculates the fraction of correctly identified brain MR images flagged as abnormal. F-score is the weighted average or harmonic mean of PPV and TPR. AUC is an effective way of quantifying the overall performance of the test. Accuracy represents the percentage of correctly classified brain MR images, including both normal and abnormal, over the total number of images. The mathematical interpretations of all these parameters are described as follows:

(20) $$\mathrm{Accuracy}=\frac{\mathrm{TP}+\mathrm{TN}}{\mathrm{TP}+\mathrm{TN}+\mathrm{FP}+\mathrm{FN}},$$

(21) $$\mathrm{TPR}=\frac{\mathrm{TP}}{\mathrm{TP}+\mathrm{FN}},$$

(22) $$\mathrm{TNR}=\frac{\mathrm{TN}}{\mathrm{TN}+\mathrm{FP}},$$

(23) $$\mathrm{PPV}=\frac{\mathrm{TP}}{\mathrm{TP}+\mathrm{FP}},$$

(24) $$F-\mathrm{Score}=2\left(\frac{\mathrm{PPV}\times \mathrm{TPR}}{\mathrm{PPV}+\mathrm{TPR}}\right),$$

(25) $$\mathrm{DSC}=\frac{2\times \left|S\cap S_{G}\right|}{\left|S\right|+\left|S_{G}\right|},$$

(26) $$\mathrm{AUC}=\frac{\mathrm{TPR}+\mathrm{TNP}}{2},$$

where S = segmented image; $S_{G}$ = ground truth; TP = true positive; FN = false negative; FP = false positive and TN = true negative.

## 4. Results and Discussion

In this section, we present experimental outcomes to demonstrate the performance of the proposed methodology. To assess the efficiency of our model, we conduct a wide range of experiments using K-FCV. Typically, it is a simple and effective method compared to other cross-validation approaches [44] and is mainly used to reduce overfitting. The selection of the K-value is a significant aspect of the classification problems. A small value of K will result in high bias, low variance, and an underfitting model. Similarly, a high value of K yields low bias, high variance, and an overfitting model. Therefore, we have chosen a moderate value for K as five to avoid this ambiguity.

### 4.1. Experimental Outcomes

This study implemented an efficient framework to identify and classify brain MR images using lightweight CNN and FL-MSCM. Primarily, we extracted brain cells from MR images to improve the accuracy of diagnosis by removing the non-brain matter using mathematical morphology and thresholding operations. Then, we employed data augmentation to enhance the model’s generalization ability. Afterward, we employed CNN model to differentiate the brain MR images as normal and abnormal. Finally, we separated the infected and non-infected tumor regions from abnormal samples using the FL-MSCM-based image segmentation framework. All these experiments were carried out on Intel (R) Core (TM) i3-5005U CPU @ 2 GHz using MATLAB 2020 and Google Colab. For a better understanding, the outcomes of the proposed methodology are separated into two phases. The first phase engages the classification results; the second phase describes the segmentation results.

#### 4.1.1. Classification Analysis

To classify brain MR images, we applied a CNN model to the skull-free augmented images. Typically, our architecture automatically tries to attain the relevant features using a series of hidden layers and learns using the back-propagation approach. During the training process, we used the cross-entropy loss function. Here, to train the model, we consider the batch size of 64 and the number of epochs of 30. In addition to that, stochastic gradient descent with momentum (SGDM) [45], Adam [46], AdaMax [46], Adagrad [47], Adadelta [48], RMSProp [49], and Nadam [50] optimizers were taken into account for minimizing the loss. Table 4 represents the parameters to be considered for optimization. 

The performance of the proposed approach on various optimization techniques using 5-FCV is presented in Table 5, Table 6, Table 7, Table 8, Table 9, Table 10 and Table 11. From the representations, we identified that Adadelta yields poor results among all other optimizers (see Table 9), especially in predicting normal brain MR images because the learning rate will become very low in the late training period. Similarly, we noted that Adam, AdaMax, and Nadam optimizers performed significantly better than others, with more than 99% accuracy on average. However, Adam optimization effectively minimizes the loss function since it slows down when converging to the local minima and minimizes the high variance. Hence, it provides better results on the suggested lightweight CNN model with 99.45% TPR, 99.80% TNR, 99.91% PPV, 99.68% F-score, 99.66% AUC, and 99.58% accuracy (see Table 6). 

The suggested methodology is compared with other well-received techniques, as illustrated in Table 12. From this, we note that the proposed diagnosis approach provides better results on the given benchmark dataset than the traditional CNN-based approaches [12,16,17,18,25,27,28,29,30]) and other machine learning frameworks. The significant advantages of the proposed method are:

1. Fewer parameters to train the model, approximately 0.35 million.
2. Minimize the overfitting problems due to the initialization of weights in the layer.
3. Significantly achieved high performance due to image data augmentation.
4. Low computational time.
5. Extraction of complex features without human intervention.

#### 4.1.2. Segmentation Analysis

The assessment of the proposed segmentation methodology is presented in Table 13, while an evaluation of the suggested approach with existing techniques is illustrated in Table 14. The outcomes of our framework are 0.96 DSC, 99.83% PPV, 99.8% TPR, 96.5% TNR, 99.82% F-score, 98.15% AUC, and 99.65 % accuracy. Based on the analysis of segmentation results (Table 14), we conclude that the proposed framework achieved remarkable performance compared to the existing techniques in terms of DSC. It must be noted that in evaluating the segmentation, higher values of DSC represent good performance. Even a small increment in this metric is remarkable and essential for clinical decisions. The reasons behind the success of the proposed segmentation methodology are:

1. Using the proposed skull-stripping process, we significantly isolate the brain tissues from non-brain matters. Due to this, the implemented approach accurately identifies brain-related diseases.
2. The proposed FL-MSCM makes each region as homogeneous as possible, with high computational efficiency, simple parameter tuning, low reduction in contrast, and image details. It is a significant advantage of the FL-MSCM.
3. The implemented approach access adequately visible edges or boundaries.

Due to the above three reasons, the segmentation method obtained a high DSC value compared to state-of-the-art approaches mentioned in Table 14.

## 5. Conclusions and Future Scope

Considering the spread of brain tumor-related cases and their impact on human life, we proposed an efficient methodology to differentiate between normal/abnormal brain MR images based on CNN and FL-MSCM. This study initially utilized the skull-stripping process to isolate extra-cranial tissues from MR images. Further, we generated augmented images using geometric transformation operators. After that, each augmented slice is fed to our lightweight CNN model to classify brain MR slices as normal and abnormal. Finally, the FL-MSCM-based automatic segmentation approach is applied to abnormal brain MR slices for identifying the region of interest (or pixels of infected organs). Based on a detailed analysis of experimental outcomes, we observed that our framework has low-computational time and achieved high performance with an accuracy of 99.58% compared to the well-received approaches due to the automatic feature learning, appropriate selection of the number of training/testing samples, effective hyper-parameter tuning, and adequately access the visible edges or boundaries from an image. Hence, anatomists can use the recommended method as a decision-making tool during clinical therapy. This paper mainly focused on binary classification (normal vs. abnormal). In the future, our work would extend to the multiclass classification of brain MR images (normal vs. sarcoma vs. glioma vs. meningioma vs. Alzheimer’s) and other medical diseases such as breast, skin, and lung cancers, etc. In addition, we would like to extend our work on real-time experimental data.

## Figures and Tables

**Table 1:** Summary of the state-of-the-art-approaches.

Reference	Methods to Be Used	Accuracy	Pros	Cons
Kale et al. [8]	LBP and SP	Accuracy = 96.17%	Significantly extract the directional details of abnormal tissues.	Performance of the model depends upon the selection of orientation bands.
Singh et al. [9]	DWT and ICA	Accuracy = 98.87%	Relatively obtain the spatial information that is useful in the classification of brain MR images.	The selection of an appropriate mother wavelet is a major challenge.
Bahadure et al. [10]	FCM and BWT	DSC = 82%	In expensive to compute and manipulate.	Relatively required large number of coefficients for approximating the smooth functions.
Gokulalakshmi et al. [11]	DWT and GLCM	Accuracy = 92.76%	Low-processing time and easy to implement.	Selection of displacement vector.
Toğaçar et al. [12]	BrainMRNet	Accuracy = 96.05%	Substantially abstract the features.	Working on low-resolution images.
Neffati et al. [13]	DWT and PCA	Accuracy = 97.02%	Reduce overfitting and improve visualization.	Loose the some significant information.
Wang et al. [14]	SWT and Entropy	Accuracy = 96.6%	Significantly highlighting the image edge features.	Irrelevant features might be extracted due to wavelet aliasing.
Arunkumar et al. [15]	K-means clustering and ANN	Accuracy = 94.07%	Woks very well on limited data.	Selection of K-value is difficult.
Toğaçar et al. [16]	CNN and hyper-column feature selection	Accuracy = 96.77%	Relatively retain the local discriminative features.	High computational time.
Chanu et al. [17]	CNN	Accuracy = 97.14 %	Less computational time.	Low performance on normal brain MR images
Lu et al. [18]	AlexNet	Accuracy = 95.71 %	Perform well on abnormal brain MR images.	Large number of parameters to be need for training.
Vishnuvarthanan et al. [19]	SOM-FKM	DSC = 47%	Faster convergence with significant accuracy.	Requires necessary and sufficient information for developing significant clusters.
Hasan et al. [20]	Modified GLCM	Accuracy = 97.8 %	Achieved remarkable accuracy and also independent on atlas registration.	Large memory requirements and computationally expensive.
Nagarathinam et al. [21]	GLCM and Morphological operations	DSC = 92%	Does not required any seed points for identification of brain MR tumor Images.	Low classification accuracy on abnormal brain MR images.
Ahmadi et al. [22]	CNN	DSC = 91%	Faster segmentation.	Heavily depends upon the network design parameters.
Toufiq et al. [23]	DWT, PCA, GLCM	Accuracy = 98 %	Minimizing the overfitting problems.	Loss of essential details due to the feature reduction process.
Ginni Garg et al. [24]	SWT, PCA, and Hybrid Ensemble classifier	Accuracy = 97.305%	Relatively improve the robustness of derived texture features.	High time complexity.
Pitchai et al. [25]	GLCM, FKM and ANN	Accuracy = 94%	Does not effect by over segmentation.	The accuracy of the ANN classifier entirely depends on the number of hidden neurons (HN).
Siyuan et al. [26]	Improved AlexNet, ELM and CBM	Accuracy = 98 %	Minimizing the overfitting problems	High computational complexity.
Mantripragada et al. [27]	AFDM and DNN	Accuracy = 96.22%	High convergence rate.	Low training speed and high complexity.
Amin et al. [28]	CNN and Nonlinear Lévy Chaotic Moth Flame Optimizer (NLCMFO)	Accuracy = 97.4%	Effective hyper-parameter tuning.	Difficulty in deterinming the ideal control parameters in NLCMFO.
Sharma et al. [29]	ResNet 50, EWS	Accuracy = 92 %	Significantly locate the boundary pixels of the tumor.	High computational time and heavily depends on batch normalization layers.
Sharma et al. [30]	VGG 19	Accuracy = 98.04%	Relatively working on a more extensive database.	Only the axial dataset of brain tumors was considered.
Haitham et al. [31]	Cascaded CNN	DSC = 85.3%	Relatively achieved good performance in a limited brain MR image database.	Required more time to train the parameters.

**Table 2:** Parameter settings of the proposed augmentation operators.

Augmentation Operator	Value
Rotation	Randomly from −30° to 30°
Translation	Translate along X (horizontal) and Y (vertical) directions with a range of [−10,10]
Reflection	Reflect randomly along X and Y-direction
Scale	Uniform scaling with a range of [0.5 to 4]
Shear	Shearing along vertical and horizontal with a range of [0° to 30°]

**Table 3:** Configurations of the suggested CNN model.

Block	Layers and Specifications	Size of the Activations	Parameters
-	Input image	224 × 224 × 3	0
-	5 × 5 Convolution with 32 filters	110 × 110 × 32	2432
	Batch norm	110 × 110 × 32	128
1	3 × 3 Convolution with 48 filters	54 × 54 × 48	13,872
	Batch norm	54 × 54 × 48	192
	3 × 3 Convolution with 48 filters	52 × 52 × 48	20,784
	Batch Norm	52 × 52 × 48	192
	1 × 1 Convolution with 48 filters	52 × 52 × 48	2352
	Batch Norm	52 × 52 × 48	192
	2 × 2 Average pooling with stride 2	26 × 26 × 48	0
2	3 × 3 Convolution with 64 filters	24 × 24 × 64	27,712
	Batch Norm	24 × 24 × 64	256
	3 × 3 Convolution with 64 filters	22 × 22 × 64	36,928
	Batch Norm	22 × 22 × 64	256
	1 × 1 Convolution with 64 filters	22 × 22 × 64	4160
	Batch Norm	22 × 22 × 64	256
	2 × 2 Average pooling with stride 2	11 × 11 × 64	0
3	3 × 3 Convolution with 128 filters	9 × 9 × 128	73,856
	Batch Norm	9 × 9 × 128	512
	3 × 3 Convolution with 128 filters	7 × 7 × 128	147,584
	Batch Norm	7 × 7 × 128	512
	1 × 1 Convolution with 128 filters	7 × 7 × 128	16,512
	Batch Norm	7 × 7 × 128	512
	2 × 2 Average pooling with stride 2	3 × 3 × 128	0
	Global average pooling	-	0
	Fully connected layer	-	258
	Trainable parameters		347,954
	Non- trainable parameters		1504
	Total parameters		349,458

**Table 4:** Parameter settings of optimizer.

Optimizer	Parameters
SGDM	α = 0.001, momentum = 0.9
Adam	α = 0.001, $\beta_{1}$ = 0.9, $\beta_{2}$ = 0.999, and ε = 10×10^−7^
Adamax	α = 0.001, $\beta_{1}$ = 0.9, $\beta_{2}$ =0.999, and ε = 10×10^−7^
Adagrad	α = 0.001 and ε = 10×10^−7^
Adadelta	α = 0.001, ε = 10 × 10^−7^ and rho = 0.95
RMSprop	α = 0.001, ε = 10×10^−7^ and rho = 0.9
Nadam	α = 0.001, $\beta_{1}$ = 0.9, $\beta_{2}$ =0.999, and ε = 10×10^−7^

Note: α represents learning rate; $\beta_{1}$, $\beta_{2}$ and ‘rho’ are the decay factors; ε is the constant for numerical stability and usually taken smaller value.

**Table 5:** Classification performance of the proposed model on SGDM optimizer.

5-FCV	Performance Metrics (%)					
	TPR	TNR	PPV	F-Score	AUC	Accuracy
1st Fold	100	99.13	99.54	99.77	99.56	99.7
2nd Fold	99.11	98.15	99.11	99.11	98.63	98.8
3rd Fold	100	95.57	97.78	98.87	97.78	98.5
4th Fold	97.85	99	99.56	98.67	98.42	98.2
5th Fold	99.56	96.15	98.27	98.91	97.85	98.5
Mean ± SD	99.3 ± 0.4	97.6 ± 0.73	98.85 ± 0.35	99.06 ± 0.19	98.45 ± 0.32	98.74 ± 0.52

Note: SD = for standard deviation.

**Table 6:** Classification performance of the proposed model on Adam optimizer.

5-FCV	Performance Metrics (%)					
	TPR	TNR	PPV	F-Score	AUC	Accuracy
1st Fold	100	100	100	100	100	100
2nd Fold	100	100	100	100	100	100
3rd Fold	97.7	100	100	98.84	98.9	98.5
4th Fold	100	100	100	100	100	100
5th Fold	99.57	98.98	99.57	99.57	99.4	99.4
Mean ± SD	99.45 ± 0.44	99.80 ± 0.2	99.91 ± 0.08	99.68 ± 0.36	99.66 ± 0.22	99.58 ± 0.29

Note: SD = for standard deviation.

**Table 7:** Classification performance of the proposed model on Adamax optimizer.

5-FCV	Performance Metrics (%)					
	TPR	TNR	PPV	F-Score	AUC	Accuracy
1st Fold	98.68	100	100	99.33	99.34	99.1
2nd Fold	98.58	100	100	99.28	99.3	99.1
3rd Fold	97.4	100	100	98.68	98.7	98.2
4th Fold	99.56	99.04	99.56	99.56	99.3	99.4
5th Fold	100	100	100	100	100	100
Mean ± SD	98.84 ± 0.45	99.80 ± 0.19	99.91 ± 0.08	99.37 ± 0.29	99.33 ± 0.21	99.16 ± 0.29

Note: SD = for standard deviation.

**Table 8:** Classification performance of the proposed model on Nadam optimizer.

5-FCV	Performance Metrics (%)					
	TPR	TNR	PPV	F-Score	AUC	Accuracy
1st Fold	100	100	100	100	100	100
2nd Fold	99.56	100	100	99.78	99.8	99.7
3rd Fold	100	89.52	95.4	97.64	97.7	96.7
4th Fold	100	100	100	100	100	100
5th Fold	99.56	100	100	99.78	99.8	99.7
Mean ± SD	99.82 ± 0.11	97.90 ± 2.1	99.08 ± 0.92	99.44 ± 0.45	99.46 ± 0.44	99.22 ± 0.63

Note: SD = for standard deviation.

**Table 9:** Classification performance of the proposed model on Adadelta optimizer.

5-FCV	Performance Metrics (%)					
	TPR	TNR	PPV	F-Score	AUC	Accuracy
1st Fold	89.57	83.60	90.43	89.99	86.6	87.38
2nd Fold	96.44	79.63	90.79	93.53	88.04	90.99
3rd Fold	99.54	80.35	90.90	95.02	89.94	93.01
4th Fold	96.10	81.37	92.12	94.07	88.74	91.6
5th Fold	99.58	58.33	85.51	92.01	78.95	87.68
Mean ± SD	96.25 ± 1.82	76.66 ± 4.63	89.95 ± 1.14	92.92 ± 0.88	86.45 ± 1.95	90.13 ± 1.1

Note: SD = for standard deviation.

**Table 10:** Classification performance of the proposed model on RMSProp optimizer.

5-FCV	Performance Metrics (%)					
	TPR	TNR	PPV	F-Score	AUC	Accuracy
1st Fold	100	100	100	100	100	100
2nd Fold	98.68	100	100	99.33	99.34	99.01
3rd Fold	87.66	99.06	99.5	93.20	93.36	91.3
4th Fold	100	80.33	89.78	94.61	90.2	92.8
5th Fold	100	100	100	100	100	100
Mean ± SD	97.268 ± 2.41	95.88 ± 3.89	97.85 ± 2.02	97.43 ± 1.46	96.58 ± 2.02	96.62 ± 1.89

Note: SD = for standard deviation.

**Table 11:** Classification performance of the proposed model on Adagrad optimizer.

5-FCV	Performance Metrics (%)					
	TPR	TNR	PPV	F-Score	AUC	Accuracy
1st Fold	98.16	100	100	99.07	99.08	98.8
2nd Fold	97.8	95.24	97.8	97.8	96.52	96.99
3rd Fold	99.54	97.34	98.65	99.09	98.44	98.8
4th Fold	100	75.96	90.16	94.82	87.98	92.5
5th Fold	97.83	95.14	97.83	97.83	96.48	96.99
Mean ± SD	98.67 ± 0.46	92.74 ± 4.28	96.88 ± 1.73	96.82 ± 0.77	97.72 ± 1.99	96.82 ± 1.15

Note: SD = for standard deviation.

**Table 12:** Classification performance of the suggested method and existing works.

Methodology	Number of Images	Data Augmentation (Yes/No)	Parameters (Millions)	Metrics (%)		
				TPR	TNR	Accuracy
MGLCM + MLP [20]	165	No	-	98.1	97.6	97.8
SW Entropy + RBF-SVM [14]	255	No	-	98.97	85	96.6
ANN [15]	230	No	-	90.9	96.78	94.07
DWT + KPCA + SVM [13]	255	No	-	100	85	97.02
AlexNet [18]	291	No	56.8	100	75	95.71
LBPSPEnerg + BPNN [8]	612	No	-	98.97	87.5	96.17
DWT + ICA + RBF-SVM [9]	240	No	-	98.97	97.68	98.87
DWT + GLCM + SVM [11]	750	No	-	99.48	60	92.76
BrainMRNet [12]	253	Yes	0.605	96	96.08	96.05
AlexNet + VGG-16 + RFE [16]	310	Yes	27.82	97.83	95.74	96.77
2D CNN [17]	309	Yes	-	100	94.11	97.14
DWT + PCA + RF [23]	181	No	-	99.2	97.8	98
SWT-GLCM-Hybrid Classifier [24]	2556	No	-	97.04	97.60	97.31
FKM-ANN [25]	-	-	-	98	99	94
AlexNet-ELM-CBM [26]	359	No	62.3	97.14	95.71	96.43
AFDF-DNN [27]	-	-	-	98.35	50	96.44
CNN [28]	694	No	-	96	98.6	97.4
Modified ResNet50 [29]	278	Yes	23.68	83	80	92
VGG-19 [30]	257	No	143	100	94.73	98.04
The Proposed (lightweight CNN)	185	Yes	0.349	99.45	99.8	99.58

**Table 13:** Performance of the suggested MR based brain tumor segmentation approach.

Sample Image	DSC	PPV	TPR	TNR	F-Score	AUC	Accuracy
1	96.46	99.76	99.94	94.41	99.85	97.17	99.71
2	94.8	99.96	99.97	94.58	99.96	97.27	99.93
3	96.05	99.85	99.85	96.03	99.85	97.94	99.71
4	99.4	99.94	99.96	99.29	99.95	99.63	99.91
5	89.04	99.8	99.77	89.81	99.78	94.75	99.57
6	88	99.86	99.73	91.53	99.79	95.63	99.59
7	99.53	99.99	99.95	99.8	99.97	99.8	99.94
8	93.16	99.37	99.53	92.23	99.45	95.88	98.98
9	87.98	99.4	99.86	81.91	99.62	90.88	99.28
10	98.58	99.82	99.98	97.51	99.89	98.74	99.81
11	99.94	100	100	99.97	99.99	99.98	99.99
12	98.82	99.9	99.99	97.87	99.94	98.93	99.89
13	97.68	99.94	99.96	97.16	99.95	98.56	99.91
14	89.43	99.95	99.15	98.76	99.55	98.96	99.14
15	95.3	99.99	99.62	99.84	99.8	99.73	99.62
16	95.61	99.82	99.9	94.44	99.86	97.17	99.73
17	91.77	100	99.74	100	99.86	99.87	99.74
18	95.6	99.21	99.46	94.78	99.33	97.12	98.84
19	97.15	99.94	99.44	99.44	99.68	99.44	99.44
20	95.6	99.37	99.83	93.26	99.6	96.55	99.27
21	97.12	99.68	99.95	95.12	99.81	97.54	99.65
22	98.49	100	99.94	100	99.97	99.97	99.94
23	96.29	99.99	99.82	99.57	99.9	99.69	99.81
24	97.22	99.98	99.64	99.75	99.81	99.7	99.65
25	88.74	99.51	99.46	89.3	99.78	94.75	99.01
26	99.56	99.98	99.98	99.56	99.98	99.77	99.96
27	96.36	100	99.79	100	99.89	99.9	99.8
28	98.28	99.99	99.9	99.69	99.95	99.8	99.9
29	99.76	100	100	99.93	99.99	99.96	99.99
30	98.88	99.97	99.97	98.85	99.97	99.41	99.94
Average	95.7	99.83	99.8	96.5	99.82	98.15	99.65

**Table 14:** Segmentation performance of the proposed and existing approaches.

Method	DSC (%)
Entropy based fuzzy clustering [19]	62
SOM [19]	37
FKM [19]	36
SOM-FKM [19]	47
BWT-SVM [20]	82
Watershed-FCM [10]	93.79
Morphological operations [21]	92
CNN [22]	91
Cascaded Net [31]	85.3
The proposed (FL-MSCM)	95.7

## Data Availability

The data presented in this study are available on request from the corresponding author. The data are not publicly available due to privacy restrictions.
